# Human Anti-V3 HIV-1 Monoclonal Antibodies Encoded by the VH5-51/VL Lambda Genes Define a Conserved Antigenic Structure

**DOI:** 10.1371/journal.pone.0027780

**Published:** 2011-12-02

**Authors:** Miroslaw K. Gorny, Jared Sampson, Huiguang Li, Xunqing Jiang, Maxim Totrov, Xiao-Hong Wang, Constance Williams, Timothy O'Neal, Barbara Volsky, Liuzhe Li, Timothy Cardozo, Phillipe Nyambi, Susan Zolla-Pazner, Xiang-Peng Kong

**Affiliations:** 1 Department of Pathology, New York University School of Medicine, New York, New York, United States of America; 2 Department of Biochemistry, New York University School of Medicine, New York, New York, United States of America; 3 Molsoft LLC, La Jolla, California, United States of America; 4 Veterans Affairs New York Harbor Healthcare System, New York, New York, United States of America; 5 Department of Pharmacology, New York University School of Medicine, New York, New York, United States of America; Istituto Superiore di Sanità, Italy

## Abstract

Preferential usage of immunoglobulin (Ig) genes that encode antibodies (Abs) against various pathogens is rarely observed and the nature of their dominance is unclear in the context of stochastic recombination of Ig genes. The hypothesis that restricted usage of Ig genes predetermines the antibody specificity was tested in this study of 18 human anti-V3 monoclonal Abs (mAbs) generated from unrelated individuals infected with various subtypes of HIV-1, all of which preferentially used pairing of the VH5-51 and VL lambda genes. Crystallographic analysis of five VH5-51/VL lambda-encoded Fabs complexed with various V3 peptides revealed a common three dimensional (3D) shape of the antigen-binding sites primarily determined by the four complementarity determining regions (CDR) for the heavy (H) and light (L) chains: specifically, the H1, H2, L1 and L2 domains. The CDR H3 domain did not contribute to the shape of the binding pocket, as it had different lengths, sequences and conformations for each mAb. The same shape of the binding site was further confirmed by the identical backbone conformation exhibited by V3 peptides in complex with Fabs which fully adapted to the binding pocket and the same key contact residues, mainly germline-encoded in the heavy and light chains of five Fabs. Finally, the VH5-51 anti-V3 mAbs recognized an epitope with an identical 3D structure which is mimicked by a single mimotope recognized by the majority of VH5-51-derived mAbs but not by other V3 mAbs. These data suggest that the identification of preferentially used Ig genes by neutralizing mAbs may define conserved epitopes in the diverse virus envelopes. This will be useful information for designing vaccine immunogen inducing cross-neutralizing Abs.

## Introduction

Human monoclonal antibodies (mAbs) against the third variable domain (V3) of the HIV-1 gp120 envelope protein derived from HIV-1 infected individuals display the ability to neutralize primary isolates representing different clades [1], [2], [3], [4], [5]. Several anti-V3 mAbs produced in our laboratory neutralized all tested neutralization-sensitive (Tier 1) pseudotyped viruses (psVs) and 30% of psVs exhibiting a less sensitive (Tier 2) phenotype [4]. Anti-V3 mAbs also protect against viral infection in experimental models [6], [7], [8] and could play a similar role when elicited by a HIV vaccine.

Anti-V3 mAbs display a broad range of cross-neutralizing activities depending on conserved elements in the V3 loop and other factors, including immunoglobulin (Ig) gene usage. A study of Ig variable genes of heavy chains (VH) used by a panel of human anti-V3 mAbs revealed a significantly altered and restricted pattern of VH gene usage when compared to other anti-HIV-1 mAbs [9], [10]. One Ig gene in particular, VH5-51, was preferentially used by 18 of 51 (35%) anti-V3 mAbs, and is not used by 44 other anti-HIV-1 mAbs specific to the CD4-binding site (CD4bs), CD4 induced antigen (CD4i) and gp41 [9]. In contrast, anti-CD4i and anti-gp41 mAbs preferentially used the VH1-69 gene segment [9], [10]. Several other studies have reported that human Abs against various pathogens also exhibit preferential VH gene usage. For example, Abs against the capsular polysaccharide of *Hemophilus influenzae* type b primarily utilize the VH3-23 gene [11], Abs against Rotavirus predominantly use the VH1-46 gene segment [12] while some human mAbs against glycoprotein gB of human cytomegalovirus are encoded by a pairing of the VH3-30 and VL kappa 3 genes [13], [14], [15].

In the context of stochastic recombination of Ig variable genes and different pairings of the heavy and light chain genes, the dominance of one particular VH gene paired in a restricted fashion with specific light chain variable genes (VL) suggests the existence of a predetermined structure of the antigen-binding site which fits to a particular epitope. To test this hypothesis, we analyzed the crystal structure of five Fabs of VH5-51/VL lambda genes encoded anti-V3 mAbs in complex with various V3 peptides.

The results confirmed our hypothesis and showed that (a) the shape of the antigen-binding site is similar in the five VH5-51/VL lambda encoded V3 mAbs and is primarily formed by the CDR H1, H2, L1 and L2 domains, (b) the majority of the key contact residues of the mAbs are the same and germline-encoded, and (c) the epitopes of these V3 mAbs have a very similar 3D structure. Furthermore, (d) a single mimotope peptide which mimics this epitope is recognized by a majority of VH5-51 anti-V3 mAbs, but not by other non-VH5-51 derived mAbs. These results suggest that identifying Ig genes preferentially used by neutralizing anti-HIV-1 mAbs has the potential to indicate the presence of conserved epitopes/antigens in diverse virus envelopes, which can then be used to design an immunogen based vaccine which induces cross-neutralizing Abs.

## Results

### VH5-51-derived human anti-V3 monoclonal antibodies

Recent analysis of the Ig variable genes coding for the heavy chains showed that the VH5-51 gene segment was preferentially used by 18 of 51 (35%) anti-V3 mAbs (Table 1) [9]. These VH5-51 V3 mAbs were generated from unrelated individuals living in the New York City area, Cameroon and India and infected with clade B, CRF02_AG and clade C, respectively (Table 1). The amino acid sequences of the VH and VL fragments of 18 VH5-51 mAbs are shown in Figure S1A and S1B, respectively. The complementarity determining regions 3 for the heavy chain (CDR H3) are different for each mAb indicating their uniqueness (Figure S1A).

**Table 1:** Human anti-V3 HIV-1 monoclonal antibodies encoded by the VH5-51 gene segment and analyzed in this study.

#	mAb^1^	Isotype	IGHV	IGLV lambda	Virus subtype^2^	Country of origin	Reference
1	257	IgG1 λ	VH5-51	1-47	B	USA	[31]
2	782	IgG1 λ	VH5-51	1-47	B	USA	[33]
3	908	IgG1 λ	VH5-51	1-47	B	USA	[33]
4	1006-15	IgG1 λ	VH5-51	1-47	B	USA	[33]
5	2219^3^	IgG1 λ	VH5-51	1-47	B	USA	[34]
6	2483^3^	IgG1 λ	VH5-51	1.47	B	USA	[35]
7	2456	IgG1 λ	VH5-51	3-25	B	USA	[34]
8	419	IgG1 λ	VH5-51	6-57	B	USA	[32]
9	838	IgG1 λ	VH5-51	3-1	B	USA	[33]
10	4085	IgG1 λ	VH5-51	1-47	CRF02_AG	Cameroon	[9]
11	2557	IgG1 λ	VH5-51	3-1	CRF02_AG	Cameroon	[35]
12	2558	IgG1 λ	VH5-51	3-1	CRF02_AG	Cameroon	[35]
13	3019	IgG1 λ	VH5-51	3-1	CRF02_AG	Cameroon	[2]
14	3694	IgG1 λ	VH5-51	3-1	H	Cameroon	[9]
15	3792	IgG1 λ	VH5-51	3-1	C	India	[9]
16	3906	IgG1 λ	VH5-51	3-1	C	India	[9]
17	4022	IgG1 λ	VH5-51	3-10	C	India	[9]
18	4025	IgG1 λ	VH5-51	3-10	non-B	India	[9]

1 All mAbs were produced from HIV-1-infected individuals using cellular methods as described [31], [36];

2 Virus subtype which infected the blood donors;

3 These two mAbs, 2219 and 2483, which are derived from the same donor, are unique (see CDR H3 sequences in Figure S1).

### Light chain genes used by VH5-51-derived anti-V3 mAbs

**Figure 1:**
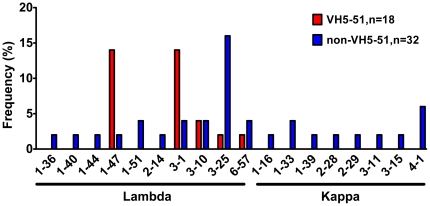
Frequency (percentage) of light chain gene segments used by VH5-51 and non-VH5-51 encoded anti-V3 mAbs. VH5-51 paired exclusively with VL lambda genes, mainly 1-47 and 3-1, while non-VH5-51 paired with 18 various VL gene segments, both kappa and lambda, with preferential usage of VL lambda 3-25. The latter paired mainly with the VH4-59 gene segment (see Table S1).

Forty-eight light chains of human anti-V3 mAbs were sequenced to determine VL gene usage. All 18 VH5-51 genes paired only with lambda variable genes and 14 out of 18 used two particular lambda genes, L1-47 (n = 7) and L3-1 (n = 7), while the remaining four mAbs used L3-10 (n = 2), L3-25 (n = 1) and L6-57 (n = 1) genes (Figure 1 and Table 1). In contrast, the 30 non-VH5-51 V3 mAbs used both VL genes with a dominance of lambda (n = 20) over kappa (n = 10) (Figure 1, Table S1).

We also analyzed the pairing of VH5-51 and VL genes on 11 Abs derived from single B cells of healthy individuals [16]. These Abs used six kappa and five lambda light chains, and each gene segment was different with the exception of two Abs paired with L1-47 (data not shown). Thus we concluded that pairing of VH5-51 gene with lambda genes in anti-V3 mAbs may reflect their restricted selection by a specific V3 epitope.

### Crystallographic analysis of the V3 epitopes recognized by VH5-51 mAbs

The specific epitope shapes within the V3 crown targeted by five VH5-51 derived anti-V3 mAbs were determined by crystal structures of their Fabs in complex with three V3 peptides representing the sequences of MN, NY5 (clade B viruses) and consensus clade A. The following structure complexes were determined: mAb 1006-15/V3_MN_ (1006/MN), mAb 2219/V3_MN_ (2219/MN), mAb 2557/V3_NY5_ (2557/NY5), mAb 2258/V3_MN_ (2558/MN), and mAb 4025/V3_ConA_ (4025/ConA). Structures of three complexes, 1006/MN, 2219/MN and 2557/NY5, were used previously to analyze the conserved elements of the V3 region [17], [18], while complexes 2558/MN and 4025/ConA were determined for this study (Table 2).

**Table 2:** Crystallography data collection and refinement statistics.

Parameter	Fab/Peptide Complex	
	2558/MN	4025/ConA
Data Collection		
Beamline	X4C, NSLS	X4C, NSLS
Space group	C2	P3_1_21
Unit cell dimensions:		
a, b, c (Å)	102.4, 73.1, 88.7	136.1, 136.1, 73.4
α, β, γ (°)	90.0, 122.0, 90.0	90.0, 90.0, 120.0
Wavelength (Å)	0.97854	0.97883
Resolution (Å)	50-1.60 (1.63–1.60)	40.0-2.0 (2.07–2.00)
Completeness (%)	99.2 (98.4)	98.5 (87.9)
Redundancy	6.1 (6.1)	8.7 (5.0)
I/σ	29.1 (4.2)	11.9 (1.2)
R_sym_ (%)^1^	5.4 (43.1)	10.6 (61.6)
Refinement		
Resolution (Å)	17.2–1.6	39.3–2.0
Number of reflections	72601	52488
R_work_ (%)	17.42	17.66
R_free_ (%)	20.30	21.34
Rmsd bond length (Å)	0.006	0.007
Rmsd bond angles (°)	1.602	1.149
Ramachandran analysis		
Favored (%)^2^	98.07	96.98
Allowed (%)^2^	1.69	3.02
Outliers (%)^2^	0.24	0.00
PDB Accession Code	3UJI	3UJJ

1 Values for the highest resolution shell are given in parentheses.

2 Actual number of residues is indicated in parentheses.

**Figure 2:**
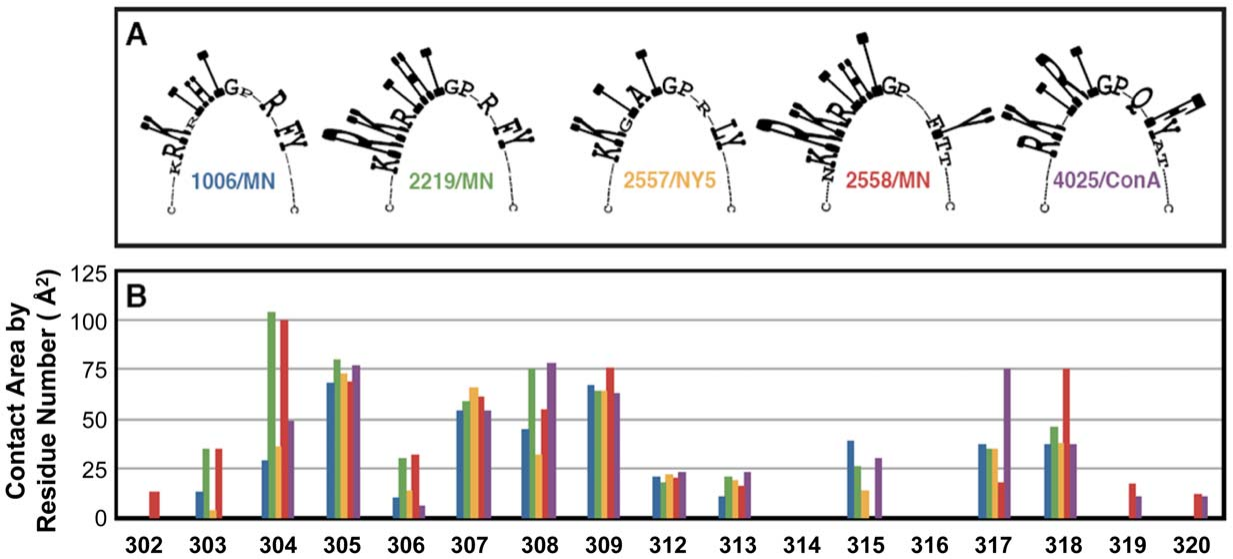
The epitopes of five VH5-51-encoded V3 mAbs. The epitopes were defined by atomic interactions of the corresponding Fab fragments with the three V3 peptides in complex. (A) Contact residues in the V3 peptides for each epitope-Fab complex of the anti-V3 mAbs. The height of each letter (abbreviations representing various amino acids) is proportional to the contact area of the V3 residue with the Fab fragment. (B) The contact area (Å^2^) for each residue interacting with Fab. The color for each complex is the same as the label in panel (A).

The epitope targeted by each mAb within the V3 loop crown was defined by calculating the atomic contacts of the residues of the V3 peptide in the crystallographic complex with that of the Fab using the ICM molecular modeling software package (Molsoft LLC, La Jolla, CA) [19]. Only residues with a contact area ≥10 Å^2^ were considered to be part of the epitope, and the calculation showed that up to 15 V3 crown residues can interact with the mAbs. Of these, seven V3 crown residues at positions P304, P305, P307, P308, P309, P317 and P318 have contacts with all five VH5-51 V3 mAbs, with the largest contact area between 25 Å^2^ to 100 Å^2^ (Figure 2A, 2B, Table 3). The remaining eight residues have minimal (average contact area below 25 Å^2^) or no contacts with some mAbs (Figure 2B, Table 3).

**Table 3:** Alignment of the V3 peptide residues at positions 302 to 320 that interact in complex with Fabs of VH5-51 encoded anti-V3 mAbs^1^.

mAb/peptide	302	303	304	305	306	307	308	309	312	313	314	315	316	317	318	319	320
1006/MN		K	**R**	**K**	R	**I**	**H**	**I**	G	P		R		**F**	**Y**		
2219/MN		K	**R**	**K**	R	**I**	**H**	**I**	G	P		R		**F**	**Y**		
2557/NY5			**K**	**K**	G	**I**	**A**	**I**	G	P		R		**L**	**Y**		
2558/MN	N	K	**R**	**K**	R	**I**	**H**	**I**	G	P				F	**Y**	T	T
4025/ConA			**R**	**K**		**I**	**R**	**I**	G	P		Q		**F**	**Y**	A	T
Mean area^2^	2	17	**64**	**73**	18	**59**	**57**	**69**	21	18	0	22	0	**40**	**47**	6	5

1 The residues of the V3 peptides with 10 Å^2^ or more buried surface area in complex with Fabs were calculated using ICM-Pro program [19]. The V3 contact residues with the largest buried surface between 25 Å^2^ to 100 Å^2^ (bold) located in the N-terminal (304, 305, 307, 308 and 309) and C-terminal (317 and 318) strands of the V3 crown.

2 Mean contact area (Å^2^) per residue.

The different Fabs were bound to recently defined elements of the V3 crown: the “arch” at the tip (β-turn) of the V3 loop (P312–P315), the “circlet” composed of regions in the N- and C-terminal β-strands of the crown (P306–P309 and P316–P317), and the “‘band” at the N- and C-terminal ends of the V3 crown (P304–P305 and P318) [17]. For the VH5-51 mAbs, the arch containing the GPGR/Q motif plays a minor role in binding (Figure 2B). Instead, there are two clusters of residues, with the largest contact area (25 Å^2^ to 100 Å^2^) in the circlet and band regions of the V3 crown that are common contact sites for all five VH5-51 derived Abs: i) R/K^P304^, K^P305^ and Y^P318^ in the band and ii) I^P307^, R/H/A^P308^, I^P309^ F/L^P317^ in the circlet (Figure 2, Table 3). Interestingly, at three positions, (P304, P308 and P317), the residues were different in the three V3 peptides in complex with the various Fabs, but the contact area in Å^2^ was similar, confirming the tolerance of anti-V3 mAbs for variation at these positions.

Thus, the specific epitopes targeted by five different, cross-reactive VH5-51 V3 mAbs have a similar 3D structure, consisting of seven key residues present in the N- and C-terminal sides of the V3 loop (Figure 2, Table 3).

### The V3 epitope defined by VH5-51 mAbs are represented by a single mimotope

We hypothesized that the remaining 13 VH5-51 anti-V3 mAbs, which were not crystallized in complex with the V3 peptides, may recognize the same or similar V3 epitope. To investigate a larger panel of anti-V3 mAbs for their recognition of this particular V3 conformation, we designed a constrained peptide mimotope which would preserve the 3D of the VH5-51 derived mAbs 2219, 2557 and 1006-15, as recently described, but would not react with other anti-V3 mAb that recognize different V3 epitopes [17]. The mimotope was designed on the basis of X-ray structures which suggested that a) the central 3 residues of the V3 crown (GPG) do not play an important role in loop recognition by VH5-51 mAbs, and b) the termini of the V3 crown are relatively close in space [17]. These observations suggested the use of a circular permutation strategy frequently used to map enzyme catalytic sites [20]. With this approach, the N- and C-termini of the V3 crown were joined via a short linker sequence, AlaSerSerPro, which creates a β-turn conformation that keeps the two strands together in the conformation observed in the complexes of VH5-51 mAbs 2219, 2557 and 1006-15. This linker was designed using a protein structure database search. The closest backbone match was located in the structure of the panthotenate kinase (PDB ID 2I7P). Simultaneously, the central β-turn at the tip of V3 was deleted and replaced with a disulfide bond that constrains the peptide to a loop. Deleting the turn assures that reactivity with other anti-V3s known to recognize the turn would be disrupted. The mimotopes thus consisted of C**QAFY** ASSP **RKSIHIG**AC, where **QAFY** represents the C-terminal flank of the V3 crown, ASSP is the linker, and **RKSIHIG** represents the N-terminal flank of the V3 crown. A crystal structure of this mimotope in complex with the Fab of VH5-51 encoded mAb 2557 revealed the same manner of interactions as shown in complex of 2557 with four different V3 peptides [17].

**Figure 3:**
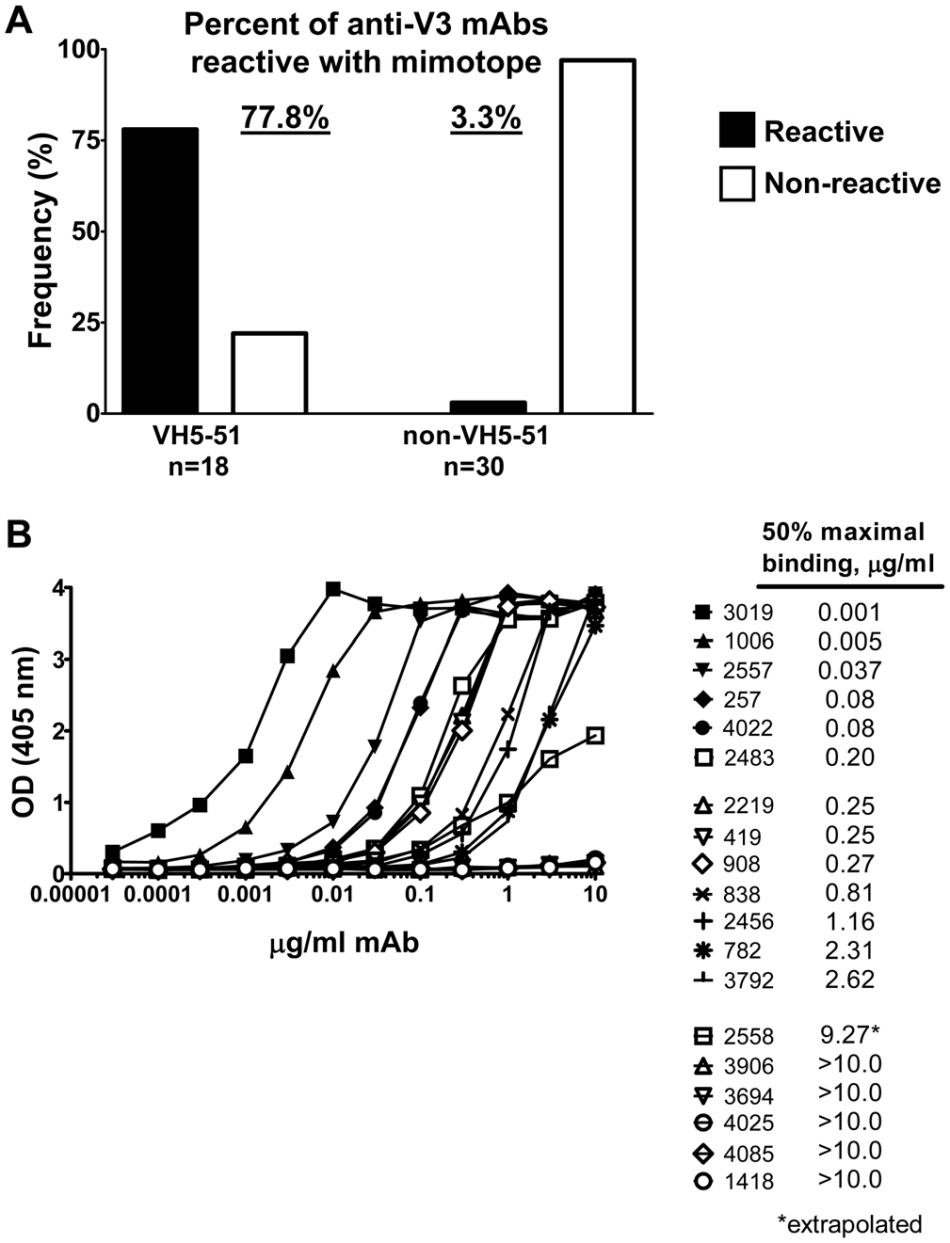
Reactivity of anti-V3 mAbs with the mimotope representing the VH5-51 epitope. (A) Binding of the VH5-51- and non-VH5-51-derived V3 mAbs to the peptide mimotope. Monoclonal Abs were tested at a concentration of 10.0 µg/ml in plates coated with biotinylated mimotope at a concentration of 1.0 µg/ml. (B) Relative affinities of mAbs binding to the mimotope as measured by 50% of maximal binding (“half-max”) to plates coated with the biotinylated mimotope at a concentration of 1.0 µg/ml. Lower half-max values reflect higher relative affinities.

This cyclic mimotope was biotinylated, immobilized on streptavidin-coated ELISA plates, and tested for reactivity with 48 anti-V3 mAbs whose gene usage was known [9]. Binding studies showed that 14 of 18 (77.8%) VH5-51 encoded mAbs were reactive with the mimotope, compared to only 1 of 30 (3.3%) non-VH5-51 mAbs (Figure 3A and Table S2). The relative affinity of 14 mimotope-reactive mAbs was determined by measuring the 50% maximal binding (“half-max”) to the mimotope (Figure 3B). All mAbs were titrated between 10 and 0.00003 µg/ml, and half-max binding values ranged from 9.3 and 0.001 µg/ml, with the lower values indicative of higher relative binding affinity. The results show that the mimotope is recognized only by V3 mAbs encoded by the VH5-51 but not by the other Ig genes.

### The CDR domains primarily determine the shape of the antigen-binding site

We hypothesized that the VH5-51 V3 mAbs may have a structurally similar shape of the antigen-binding site (ACS), as they recognize uniform V3 epitope. This was confirmed by the crystallographic analysis of five Fabs (1006-15, 2219, 2557, 2558 and 4025) in complex with three different V3 peptides (MN, NY5 and consensus A).

**Figure 4:**
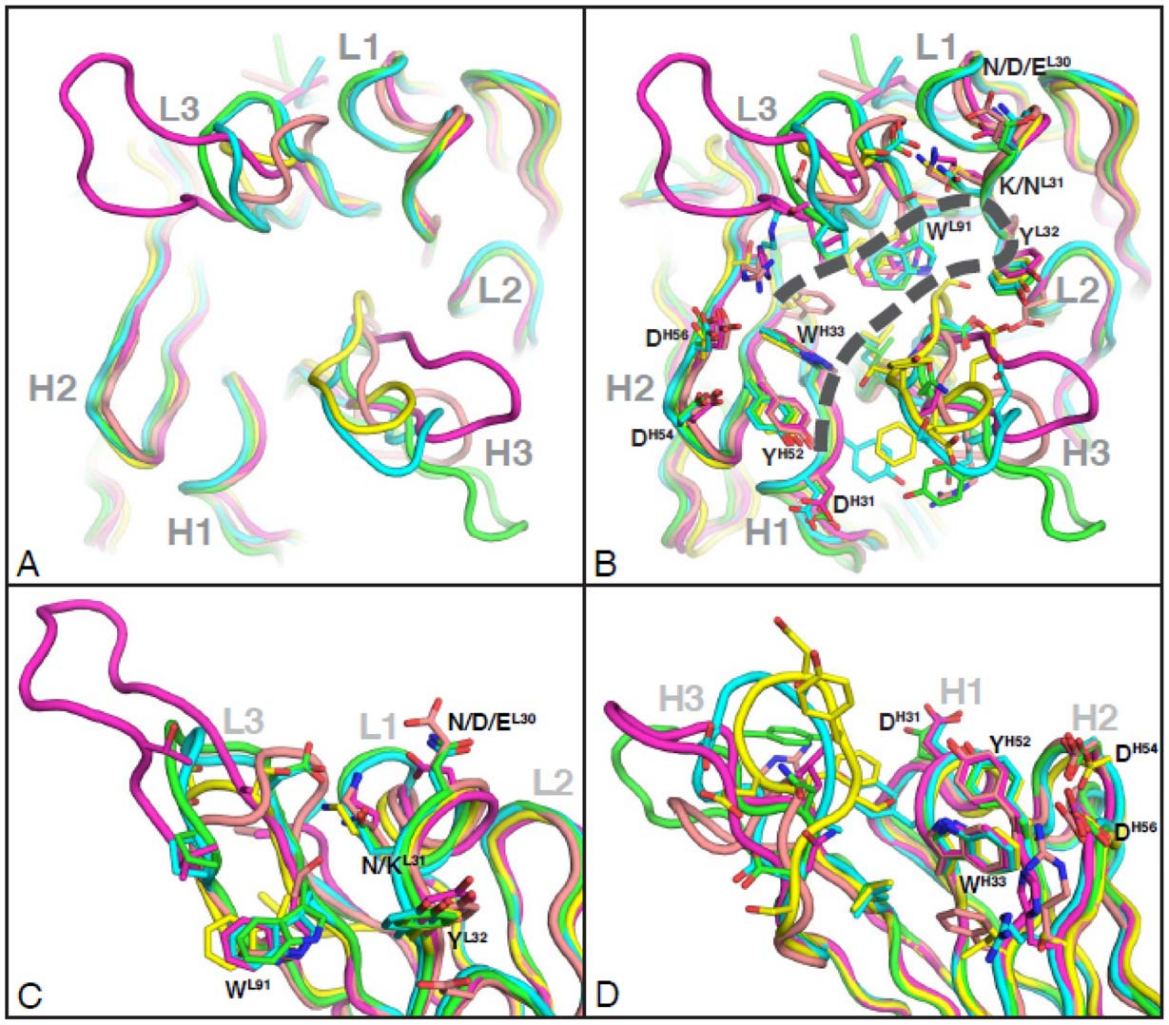
Structural similarity of the antigen-binding sites of VH5-51 encoded anti-V3 mAbs. (A) A structural superimposition of the Cα atoms of the Fab variable domains of complexes 1006/MN (green), 2219/MN (cyan), 2557/NY5 (magenta), 2558/MN (yellow), and 4025/ConA (salmon). Note the domains of CDR H1, H2, L1 and L2 superimposed well, while that of CDR L3 and H3 is more structurally divergent. (B) Same superimposition as that in panel (A) but with the key contact residues of the mAbs labeled (all contact residues are shown in Tables 4 and 5). The position of the V3 is illustrated as a dashed line. (C) and (D) Structural details of the antigen combining sites of the light (C) and heavy chains (D) in the five complexes.

Structural alignments of the variable fragment of the heavy and light chains of five VH5-51 derived Fabs in complex with V3 peptides revealed that the CDRs H1, H2, L1 and L2 loops superimpose well with a root mean square deviation (RMSD) of <1 Å (Figure 4A). Each of the CDR H3 regions of the five mAbs, however, has a different structure due to their unique sequences and do not superimpose at all (Figure 4A, 4B, 4D). CDR L3 domains has less diversity but they are still not well aligned especially for that of 2557 whose L3 is considerably longer than the others (Figure 4A, 4B, 4C).

The binding site is shaped like a cradle in which the V3 peptide, in the form of a β-hairpin, is bound (Figure 4B). The binding site is formed by (a) the CDR L1 loop interacting with the β-turn in the V3 arch, (b) the CDR L3 interacting with hydrophobic side of the circlet, (c) the CDR H3 interacting with the hydrophilic side of the V3 circlet, and (d) CDRs H1 and H2 interacting with the C- and N-termini of the V3 crown (the V3 band) (Figure 4B and [17].

Thus, the strong structural commonality in the antigen-binding site of all five crystallized mAbs is determined primarily by the CDR H1, H2, L1 and L2 domains. Upon binding of the Fabs to V3 peptides, only the CDR H3 domain was seen to move towards the antigen, while the remaining CDR domains are stable and exhibit the same backbone conformation as in unbound Fab (X-P. Kong et al., unpublished data). The differences in binding affinity to a designed mimotope (Figure 3B) and in contact maps related to mAb specificity for diverse viral epitopes results largely from differences in the structure of CDR H3, and less so L3.

### The germline-encoded CDR residues of VH5-51 V3 mAbs play key roles in antigen-antibody interactions

We speculated that the similar shape of the antigen-binding site of VH5-51 anti-V3 mAbs implied that the residues interacting with the V3 peptide are at the same position for each mAb. Crystallographic analysis confirmed our hypothesis, with the key contact residues in the CDR H1 and H2 regions in all five mAbs; they include W^H33^ in H1 (with some variations at positions H31 and H32) and Y^H52^, D^H54^ and D^H56^ in H2 (Figure 4B, 4D and Table 4). All of these contact residues are also present at the corresponding positions in the sequence of the germline VH5-51 gene which is VH genomic precursor for these mAbs (Table 4).

**Table 4:** The key contact residues in the CDRs of five anti-V3 mAbs in complex with V3 peptides^1^.

mAb/peptide	CDR H1	CDR H2	CDR H3	Ref.
1006/NY5	GYTFTDHW	IYPGDSDT	AR**L**HYSDRSGS**Y**FNDVFHM	[17]
2219/MN	GYSFSDHW	FYPGDSDS	ARLGGDYEDSGADAFDF	[18]
2557/MN	GYNFLDSW	IYPDDSDA	TRLYLFEGAQSSNAFDL	[17]
2558/MN	GYSFSNYW	VYPD**D**S**D**S	ARLGFEGDYSGSFFDY	This study
4025/ConA	GYSFSSYW	IYPA**D**S**D**T	AILGFWGANRGGGGMDV	This study

1 The contact residues (bold) of mAbs with more than 10 Å^2^ buried surface area in complex with V3 peptide were calculated using ICM program [19]. The majority of these residues (bold, underlined) in CDR H1 and H2 are also present in the corresponding VH5-51 germline gene.

Similarly, the contact residues in the CDR L1 of light chains are also germline-encoded, although they differ since three VL genes were used by five VH5-51 mAbs. The two residues in mAbs 1006-15 and 2219–N^L31^ and Y^L32^, the three residues in mAbs 2557 –D^L30^, K^L31^ and Y^L32^, and the two residues in mAbs 2558 and 4025–K^L31^ and Y^L32^ which reacted with the V3 peptides, are present in the corresponding germline sequences of lambda light chain genes (Figure 4B, 4C and Table 5). Only contact residues N^L30^ in mAbs 1006-15 and 2219, and E^L30^ in mAb 4025 (Table 5) are mutated from germline gene. In terms of CDR L2, there is only one contact residue (E^L51^) among the five mAbs which is also germline-encoded and occurs in mAb 4025 (Table 5).

**Table 5:** The key contact residues in the CDRs of the variable light chains of the VH5-51 derived anti-V3 mAbs in complex with the V3 peptides^1^.

Germline	mAb/peptide	CDR L1	CDR L2	CDR L3	Ref.
VL1-47	1006/MN	SSNIE**NNY**	RDD	AS**W**D**D**SRGGPDYV	[17]
VL1-47	2219/MN	SSNVE**NNY**	RND	AA**W**D**D**SR**GGP**DWV	[18]
VL3-1	2557/NY5	KLD**DKY**	QDF	QA**W**D**A**STGVSGGGTKLTVL	[17]
VL3-1	2558/MN	ILGD**KY**	EDT	QA**W**D**ST**LGVV	This study
VL3-10	4025/ConA	ALP**EKY**	**E**DS	YS**T**NSG**GT**FFV	This study

1 In a similar fashion as in Table 4 the contact residues (bold) are those which have more than 10 Å^2^ buried surface area in complex with the V3 peptide. Those contact residues that are present in the corresponding germline sequence are bold and underlined. The CDR L3, in contrast to CDR H3, is encoded in substantial part by VL germline gene, and the residues interacting with the peptide are also present in the corresponding germline.

The contact residues in CDR H3 in five crystallized Fabs are all different, ranging from two to eight residues (Table 4). Furthermore, their germline origin is inconclusive due to deletion and/or insertion of nucleotides during Ig gene recombination process. The CDR L3 is less diverse than the H3 region which is made up by two germline genes (VL and JL) and only few non-templated nucleotides are added.

The presence of the key contact residues in the four CDR domains (H1, H2, L1 and L2) at the same position and which are germline-encoded, except of those located in the CDR H3, suggest that the antigen-binding site has conserved backbone conformation.

### Peptide in complex with Fab adapt the shape of the antigen-binding site

**Figure 5:**
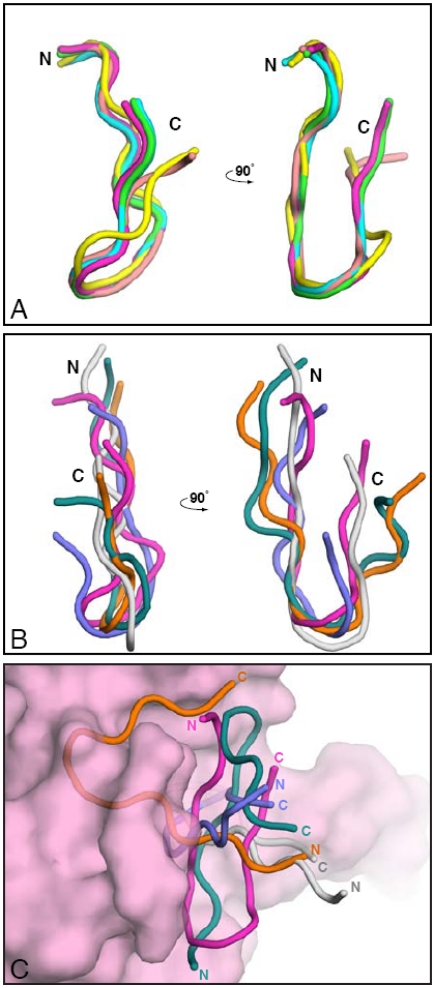
Structures of V3 peptides in complex with the VH5-51 and non-VH5-51 anti-V3 mAbs. (A) Structural superimposition of the Cα atoms of the V3 peptides in the complexes with five VH5-51 mAbs: 1006/MN (green), 2219/MN (cyan), 2557/NY5 (magenta), 2558/MN (yellow), and 4025/ConA (salmon). (B) For comparison, a structural superimposition of the V3 peptides complexed with non-VH5-51 mAbs: 537/MN (grey), 447/W2RW020 (violet), 3074/MN (orange), and 268/MN (teal); the complex of Fab with V3 peptide, 2557/NY5 (magenta), is retained for comparison. (C) V3 peptides in the various crystals noted in B after superimposing the Fab structures onto that of Fab 2557 (shown as a semi-transparent surface). Note the drastically different ways of binding V3 for the non-VH5-51 mAbs.

The V3 peptides with the sequence of MN, NY5 clade B viruses and consensus clade A and in complex with Fabs share an almost identical backbone structure shown by close superimposition of their Cα atoms (Figure 5A). There is a slight difference in the superimposition of two peptides, MN and consensus A, in complex with Fab 2558 and 4025, respectively, which have different conformations of their carboxyl terminal end due to different residues interacting with the peptides (Figure 5A, Figure S2). Since mAb 4025 (as well as mAbs 3694, 3906 and 4085) do not react with the mimotope (Table S2), they may represent a subfamily of VH5-51 encoded V3 mAbs.

This is in contrast with the backbone conformations of the V3 peptides in complex with Fabs encoded by non-VH5-51 genes (Figure 5B). As shown, the V3 peptides in complex with Fabs of non-VH5-51 anti-V3 mAbs–537 (VH3-7, VL2-14) complexed with MN, 447 (VH3-15, VL1-51) complexed with W2R020, 268 (VH4-59, L3-25) complexed with MN, and 3074 (VH4-59, VL1-51) complexed with MN–do not superimpose well (Figure 5B) [17], [21]. The different backbone conformations of the V3 peptides were particularly evident when all Fabs of non-VH5-51 V3 mAbs were aligned to mAb 2557 in the 2557/NY5 complex. As shown in Figure 5C, the V3 peptides are adapted to the shapes of the antigen-binding sites encoded by the different VH and VL paired genes, resulting in different backbone conformation of the bound V3 peptides.

From this data, it is apparent that the backbone conformation of the V3 peptide in complex with the Fab is fully adapted to, and imprints, the shape of its antigen-binding site which is encoded by particular pair of Ig genes.

## Discussion

Restricted usage of Ig gene segments is rarely observed among Abs due to the exceptional variability resulting from Ig gene rearrangements and VH and VL gene pairing. While the mechanism of preferential Ig genes selection, when it occurs, is not clear, we have concluded on the basis of our data that the VH5-51/VL lambda genes preferentially used by anti-V3 mAbs are related to a particular conserved epitope in the V3 region of the HIV-1 envelope.

The crystal structures of five VH5-51/VL lambda derived V3 mAbs in complex with V3 peptides revealed that each Fab interacted with key residues at the same seven positions in the crown of the V3 loop, despite the fact that the amino acids at these positions could vary. The observation that mAbs derived from unrelated individuals and infected with disparate HIV-1 strains use the same human Ig variable segment, react with the same key residues, and recognize a nearly identical overall conformation in the V3 crown suggest that while V3 is variable in sequence and structurally flexible, a common structure(s) is retained across strains. Our results further indicate that the mAbs utilizing the VH5-51/VL lambda genes possess similarly shaped antigen-binding sites that are highly suitable for the recognition of this common and conserved V3 conformation. Thus, preferentially used Ig genes indicate that antibodies encoded by these genes recognize a conserved epitope which can be used for design of HIV vaccine immunogen.

The conserved shape of the binding sites is reflected in the identical lengths of three CDRs, H1, H2 and L2, and minimal variation in the CDR L1 (Figure S1B). The lengths of the CDR loops contribute to the structure of the antigen-binding site [22], and if the lengths are almost identical, then the backbone conformation of the binding sites is highly comparable. Indeed, the conserved cradle shape and the four CDRs comprising this cradle (H1, H2, L1 and L2) have the same superimposed backbone conformation (Figure 4). Generally, the CDR loops, with the exception of H3 and L3, have a very limited number of canonical conformations which support a conserved structure of the binding site [23], [24].

The antigen-binding site of the VH5-51 derived V3 mAbs has a very stable backbone conformation which was not changed when Fabs were complexed with different V3 peptides. A similarly stable structure of the binding site was recently observed in a mAb against the hepatitis B virus [25]. The shape of the binding site was not changed in the peptide-bound Fab compared to the uncomplexed Fab, with the exception of CDR H3 which adapted to the antigen by changing its conformation upon complexing with the peptide. Similarly, in murine anti-V3 mAbs, only CDR H3 changed conformation upon peptide binding, with additional rotation of the VL region relative to the VH chain [25], [26]. In our preliminary structural studies of free and peptide-bound VH5-51 anti-V3 Fabs, only the CDR H3 domain changed its backbone conformation upon peptide binding, while the shape of the binding site was stable (data not shown).

The CDR H3 domain has the special function of determining the fine specificity in each antibody [27]. The structure of H3 is likely a result of affinity maturation, as it had different sequences, lengths and conformations. Previously we observed that three VH5-51 encoded mAbs (2219, 2557 and 2558) neutralized different numbers of pseudotyped viruses, especially resistant Tier 2 viruses, which might depend on differences in affinity and/or specificity [4]. While all three mAbs share conserved structure of the binding sites (Figure 4A), the CDR H3 loops are quite distinct (Table 4) and may contribute to the differences in neutralizing activity. Thus, the general structure of the binding site, as determined by the non-H3 CDRs, appears to mediate the recognition of the overall shape of the antigen, while each CDR H3 domain may contribute to Ab affinity and/or fine specificity; this would be consistent with previous results [27], [28].

We hypothesize that the structure of the antigen-binding sites in the naïve B cell germline-encoded B cell receptor and in the affinity-matured mAbs are similar, based on the observation that the key contact residues in the heavy and light chains of crystallized Fabs are germline-encoded (Table 4 and 5). We further speculate that a mimotope which specifically binds to 14 of 18 VH5-51 encoded anti-V3 mAbs, but is not reactive with non-VH5-51 V3 mAbs, binds to the receptor encoded by the VH5-51/lambda genes on the naïve B cells. Consequently, these B cells may be selected and stimulated to produce Abs specific for the relatively conserved V3 epitopes upon immunization with a mimotope-based immunogen.

We have shown that the V3 sequence-fusion proteins used as boosting immunogens after gp120 DNA priming were immunogenic and induced neutralizing Abs in rabbits [29]. Further experiments in rabbits have demonstrated that one of immunogens containing the V3 sequence recognized by mAb 2219, which is encoded by VH5-51 and VL1-47 genes, elicited cross-clade neutralizing activity [30] predicting that a mimotope-based immunogen has potential to induce V3-specific Abs with neutralizing activity.

Collectively, we have observed in this study that the preferential usage of the VH5-51 gene paired with VL lambda genes in a restricted manner forms the antigen-binding site which allows recognition of a conserved V3 epitope. This data suggest that identification of dominantly used Ig genes by antibodies, including neutralizing anti-HIV-1 mAbs, may allow for the discovery of novel conserved epitopes. It may have practical implications for the design of a vaccine immunogen targeting antigenically diverse pathogens such as HIV-1.

## Materials and Methods

### Ethics Statement

The study was approved by the New York University School of Medicine Institutional Review Board. All subjects signed written approved informed consent forms prior to participating in the study.

### Human anti-V3 mAbs

Forty-eight human anti-V3 HIV-1 mAbs were tested in this study. Eighteen mAbs are encoded by the VH5-51 gene segment (Table 1) and were selected from the panel of anti-V3 mAbs studied for Ig gene usage in the previous study [9]. Many of these were previously shown to neutralize HIV pseudoviruses and/or primary isolates from multiple subtypes and, thus, are considered to be cross-strain and cross-clade reactive [2], [9], [31], [32], [33], [34], [35]. All mAbs were produced in our laboratory by cellular methods via fusion of heteromyeloma cells with Epstein-Barr virus transformed PBMCs derived from individuals infected with clade B and non-clade B HIV-1; the resulting heterohybridoma cell lines were cloned by limiting dilution to monoclonality as previously described [31], [32], [36]. This method preserves the cognate pairing of the heavy and light chain genes and the mAbs thus accurately represent portions of the human B cell repertoire. Protein A or G purified mAbs were used for immunochemical and crystallographic analyses. The VH and VL sequences are available at GenBank (accession nos. M67504, EU794410, EU794414-17, EU794424, EU794427-28, EU794430, EU794434, EU794437, EU794441-3 and HQ890341–HQ890361).

### Peptides

Two 23-mer V3 peptides representing the sequences of clade B MN (^301^ YNKRKRIHIGPGRAFYTTKNIIG ^325^) and consensus clade A (^301^ NNTRKSIRIGPGQTFYATGDIIG ^325^) [17], [33] were used to make complexes with the Fab fragments of mAbs 2558 and 4025, respectively. The V3 peptides MN and NY5 (^301^ NNTKKGIAIGPGRTLYAREK ^322^) were used for crystallization of 1006-15, 2219 and 2557 as previously described [17]. A mimotope, CQAFYASSPRKSIHIGAC, mimicking the specific V3 epitope recognized by the VH5-51 derived human mAbs that contains elements of the N- and C- β-strands of the V3 crown connected by a linker sequence (ASSP), with the GPGR motif replaced by a disulfide bridge, was previously described [17].

### RT-PCR amplification of the Ig variable region of the light chain genes

The messenger RNA was extracted from hybridoma cell lines producing human anti-V3 mAbs and reverse transcribed into cDNA using oligo dT primer. A homopolymeric dCTP tail was added to the 3′ end of the total cDNA with terminal deoxynucleotidyl transferase. The variable domain of the lambda light chain genes (VL) was amplified from poly-C tailed cDNA by PCR using deoxyinosine-containing anchor primer as forwards primer (5′RACE Abridged Anchor primer, 5′-GGC CAC GCG TCG ACT AGT ACG GGI IGG GII GGG IIG-3′) (Invitrogen) and gene specific primer as backwards primer located in constant region of κ (5′-AAC ACT CWY YCC TGT TGA AGC TCT T-3′) or λ (5′-CAC TGT CTT CTC CAC GGT GCT CCC TTC-3′).

PCR amplification was performed using a cycling program of 2 min at 94°C, 35 cycles of 60 s at 94°C, 60 s at 55°C, and 120 s at 72°C, followed by 7 min at 72°C. Ethidium bromide-stained 0.8% agarose gels were used to visualize the PCR products. The bands of the appropriate size were excised and cleaned with GeneElute Minus EtBr Spin Column (Sigma) then were cloned into 2.1-TOPO TA cloning (Invitrogen).

For each chain, 6 to 12 independent clones were screened. The plasmids with the appropriate inserts were sequenced in both directions using the M13 primers. All sequencing reactions were performed at the Macrogen, Rockville, MD. The sequence data were analyzed using Pregap4, BioEdit software and the International ImMunoGene Tics (IMGT) information system (http://imgt.cines.fr).

### ELISA

A standard ELISA was used to determine the binding of mAbs to the mimotope as previously described [33]. The biotinylated mimotope was immobilized on streptavidin-coated plates (StreptaWell, Roche), blocked with 2% BSA in phosphate-buffered saline (PBS), and then incubated for 1.5 h at 37°C with human mAbs at concentrations ranging from 0.00003 to 10 µg/ml. The plates were washed, and the bound mAbs were detected by incubation with alkaline phosphate-conjugated goat anti-human IgG (Fc) (Zymed, San Francisco, CA) for 1.5 h at 37°C. After washing, the substrate was added for 30 min and the plates were read at 410 nm. The relative affinity of mAb binding to mimotope was assessed by measuring the concentration of mAb that gave 50% maximal binding; it was calculated by linear interpolation when the binding reached the saturation level.

### X-ray crystallography

Crystallization of Fabs in complex with V3 peptides was carried out to obtain the atomic structures of the antigen combining sites of the mAbs and their relationship to VH and VL gene usage. Briefly, Fab of each mAb was prepared by papain digestion followed by purification using Protein A affinity columns (GE Healthcare) and size exclusion chromatography [37]. Excess epitope peptide was added to the Fab solution and the Fab/V3 mixture was concentrated to 10–30 mg/ml for crystallization. Standard vapor diffusion techniques were used with sitting or hanging drop trays [21]. X-ray diffraction data were collected at the National Synchrotron Light Source at Brookhaven National Laboratory. The crystal structures were solved by the molecular replacement method using information on the sequence of the Ig heavy and light chains determined in our laboratory and from known Fab models using CNS [38] or MOLREP [39]. The structure refinement was carried out using the CNS or REFMAC [40].

## Supporting Information

Figure S1 Amino acid sequences of the variable fragment of the heavy chains (A) and light chains (B) of 18 human anti-V3 mAbs encoded by the VH5-51 gene segment. The sequences are aligned with their corresponding germlines (the sequences of the heavy chains were aligned only to one germline sequence of IGHV5-51*01 according to IMGT system). Kabat numbering is used along with the IMGT CDR domain definitions. Insertion codes are shown as lowercase letters in the numbering line, following the “base” residue number for that insertion (e.g. the first “a” in the heavy chain figure, which follows residue 52, is for residue 52A). For CDRs H3 and L3, insertion codes are represented as “x” to preserve the alignment. Dots indicate identity, while letters indicate substitutions in the heavy and light chains. CDR H3 and Framework 4 are not aligned to germlines.
(TIF)

Figure S2 Structures of light chains, heavy chains, and the V3 peptides in the V3-Fab complexes. The key contact residues of the Fabs in the light and heavy chains are labeled and numbered according to the standard Kabat numbering scheme.
(TIF)

Table S1 Immunoglobulin gene usage for variable light chain of non-VH5-51 anti-V3 mAbs.
(DOC)

Table S2 ELISA reactivity of VH5-51 and non-VH5-51 encoded anti-V3 mAbs with cyclic biotinylated mimotopes^1^.
(DOC)
