# Supplementary figures and images for: Human Anti-V3 HIV-1 Monoclonal Antibodies Encoded by the VH5-51/VL Lambda Genes Define a Conserved Antigenic Structure
Source: PLoS One. 2011 Dec 2;6(12):e27780. doi: 10.1371/journal.pone.0027780 (PMC3229485; doi:10.1371/journal.pone.0027780)

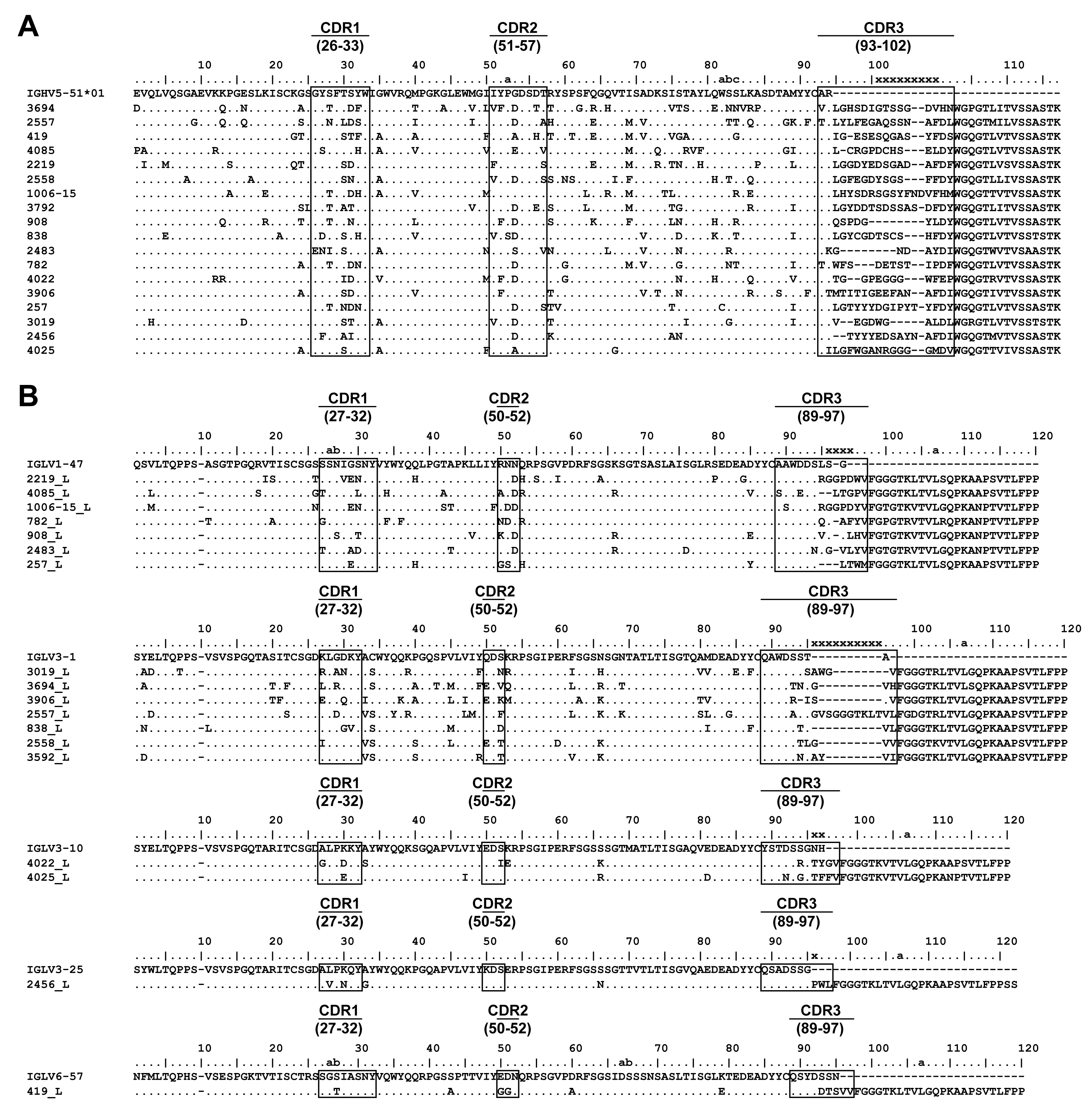

Supplement: Figure S1 — Amino acid sequences of the variable fragment of the heavy chains (A) and light chains (B) of 18 human anti-V3 mAbs encoded by the VH5-51 gene segment. The sequences are aligned with their corresponding germlines (the sequences of the heavy chains were aligned only to one germline sequence of IGHV5-51*01 according to IMGT system). Kabat numbering is used along with the IMGT CDR domain definitions. Insertion codes are shown as lowercase letters in the numbering line, following the “base” residue number for that insertion (e.g. the first “a” in the heavy chain figure, which follows residue 52, is for residue 52A). For CDRs H3 and L3, insertion codes are represented as “x” to preserve the alignment. Dots indicate identity, while letters indicate substitutions in the heavy and light chains. CDR H3 and Framework 4 are not aligned to germlines. (TIF) [file pone.0027780.s001.tif]

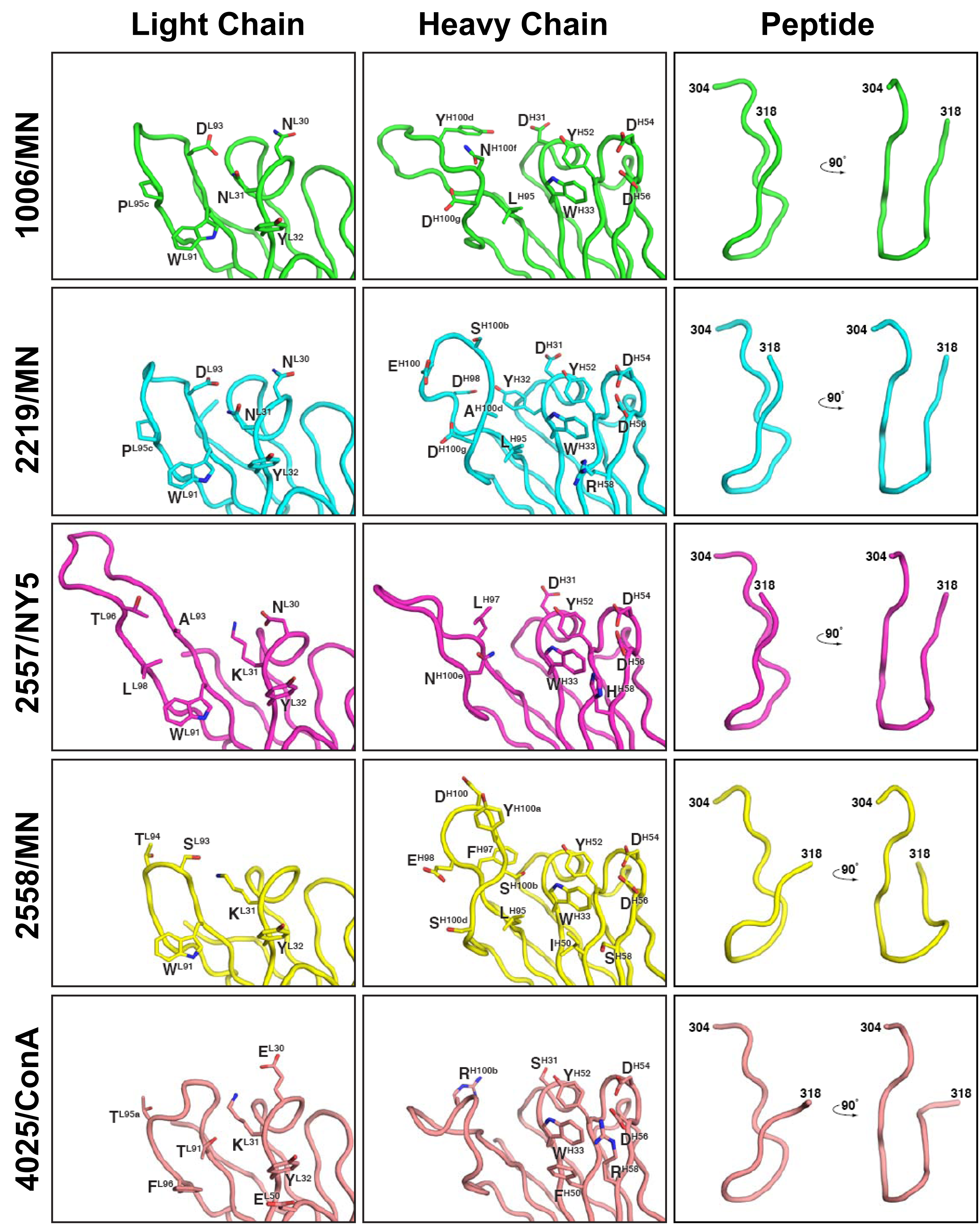

Supplement: Figure S2 — Structures of light chains, heavy chains, and the V3 peptides in the V3-Fab complexes. The key contact residues of the Fabs in the light and heavy chains are labeled and numbered according to the standard Kabat numbering scheme. (TIF) [file pone.0027780.s002.tif]
