# Supplementary material for: Human Anti-V3 HIV-1 Monoclonal Antibodies Encoded by the VH5-51/VL Lambda Genes Define a Conserved Antigenic Structure
Source: PLoS One. 2011 Dec 2;6(12):e27780. doi: 10.1371/journal.pone.0027780 (PMC3229485; doi:10.1371/journal.pone.0027780)
Supplement: Table S1 — Immunoglobulin gene usage for variable light chain of non-VH5-51 anti-V3 mAbs. (DOC) [file pone.0027780.s003.doc]

**Table S1.** Immunoglobulin gene usage for variable light chain of non-VH5-51 anti-V3 mAbs

| # | mAb^1^ | Virus sub^2^ | IGHV | IGLV |
| --- | --- | --- | --- | --- |
| 1 | **311-11** | B | 1-3 | L3-10 |
| 2 | **1334** | B | 1-3 | L3-10 |
| 3 | **3791** | C | 1-18 | K1-33 |
| 4 | **4121** | C | 1-18 | L1-44 |
| 5 | **3697** | G | 1-24 | L6-57 |
| 6 | **391/5** | B | 1-24 | K1-16 |
| 7 | **1027-15** | B | 1-f | L3-1 |
| 8 | **3869** | non-B | 1-f | L3-1 |
| 9 | **3224** | CRF09_cpx | 1-f | L1-40 |
| 10 | **2191** | B | 1-f | L1-47 |
| 11 | **2412**^3^ | B | 2-5 | L3-25 |
| 12 | **694/8** | B | 2-5 | L3-25 |
| 13 | **537** | B | 3-7 | L2-14 |
| 14 | **3402** | B | 3-7 | K4-1 |
| 15 | **447-52D** | B | 3-15 | L1-51 |
| 16 | **2601** | CRF13_cpx | 3-30 | L3-25 |
| 17 | **504**^3^ | B | 3-30 | K2-29 |
| 18 | **1324E** | CRF_AE | 3-30 | K3-11 |
| 19 | **3904** | C | 3-30 | K1-39 |
| 20 | **2424** | B | 3-53 | K2-28 |
| 21 | **412** | B | 3-33 | K4-1 |
| 22 | **418** | B | 3-53 | K4-1 |
| 23 | **268** | B | 4-59 | L3-25 |
| 24 | **2182** | CRF02_AG | 4-59 | L3-25 |
| 25 | **453** | B | 4-59 | L3-25 |
| 26 | **1108** | B | 4-59 | L3-25 |
| 27 | **386** | B | 4-59 | L3-25 |
| 28 | **2442** | B | 4-59 | L1-36 |
| 29 | **3074** | CRF02_AG | 4-59 | L1-51 |
| 30 | **3881** | CRF02_AG | 4-59 | K3-15 |

^1^All mAbs were produced from HIV-1-infected individuals using cellular methods as described [29];  ^2^Subtype of the donor’s infecting virus. Subtype B is presumed as the donors are living in NYC area. The non-B subtypes were determined by sequencing the envelope proteins; ^3^These mAbs are derived from one patient but each has a unique CDR H3 sequence.
