# Supplementary material for: Human Anti-V3 HIV-1 Monoclonal Antibodies Encoded by the VH5-51/VL Lambda Genes Define a Conserved Antigenic Structure
Source: PLoS One. 2011 Dec 2;6(12):e27780. doi: 10.1371/journal.pone.0027780 (PMC3229485; doi:10.1371/journal.pone.0027780)
Supplement: Table S2 — ELISA reactivity of VH5-51 and non-VH5-51 encoded anti-V3 mAbs with cyclic biotinylated mimotopes1. (DOC) [file pone.0027780.s004.doc]

**Table S2. ELISA reactivity of VH5-51 and non-VH5-51 encoded anti-V3 mAbs with cyclic biotinylated mimotopes1**

| # | mAb | IGHV | O.D. |  | # | mAb | IGHV/family | O.D. |
| --- | --- | --- | --- | --- | --- | --- | --- | --- |
| 1 | **1006-15** | 5-51 | **3.7** |  | 1 | **268** | 4-59 | 0.1 |
| 2 | **2219** | 5-51 | **3.6** |  | 2 | **386** | 4-59 | 0.1 |
| 3 | **419** | 5-51 | **3.5** |  | 3 | **453** | 4-59 | 0.1 |
| 4 | **2483** | 5-51 | **3.4** |  | 4 | **1108** | 4-59 | 0.3 |
| 5 | **257** | 5-51 | **3.8** |  | 5 | **2182** | 4-59 | 0.1 |
| 6 | **908** | 5-51 | **3.5** |  | 6 | **2442** | 4-59 | 0.1 |
| 7 | **782** | 5-51 | **3.5** |  | 7 | **3074** | 4-59 | 0.1 |
| 8 | **2456** | 5-51 | **3.2** |  | 8 | **3881** | VH3 | 0.1 |
| 9 | **838** | 5-51 | **3.4** |  | 9 | **412** | VH3 | 0.1 |
| 10 | **3019** | 5-51 | **3.7** |  | 10 | **418** | VH3 | 0.1 |
| 11 | **2557** | 5-51 | **3.9** |  | 11 | **447** | VH3 | 0.1 |
| 12 | **4022** | 5-51 | **3.0** |  | 12 | **504** | VH3 | 0.1 |
| 13 | **3792** | 5-51 | **3.5** |  | 13 | **537** | VH3 | 0.1 |
| 14 | **2558** | 5-51 | **1.8** |  | 14 | **1324E** | VH3 | 0.1 |
| 15 | **3694** | 5-51 | 0.1 |  | 15 | **2424** | VH3 | 0.1 |
| 16 | **3906** | 5-51 | 0.1 |  | 16 | **2601** | VH3 | 0.1 |
| 17 | **4025** | 5-51 | 0.1 |  | 17 | **3402** | VH3 | 0.1 |
| 18 | **4085** | 5-51 | 0.1 |  | 18 | **3904** | VH3 | 0.1 |
|  |  |  |  |  | 19 | **311** | VH1 | 0.1 |
|  |  |  |  |  | 20 | **391-5** | VH1 | 0.1 |
|  |  |  |  |  | 21 | **1027-15** | VH1 | **0.9** |
|  |  |  |  |  | 22 | **1334** | VH1 | 0.1 |
|  |  |  |  |  | 23 | **2191** | VH1 | 0.1 |
|  |  |  |  |  | 24 | **3224** | VH1 | 0.1 |
|  |  |  |  |  | 25 | **3697** | VH1 | 0.1 |
|  |  |  |  |  | 26 | **3791** | VH1 | 0.1 |
|  |  |  |  |  | 27 | **3869** | VH1 | 0.1 |
|  |  |  |  |  | 28 | **4121** | VH1 | 0.1 |
|  |  |  |  |  | 29 | **2412** | 2-5 | 0.1 |
|  |  |  |  |  | 30 | **694/98** | 2-5 | 0.1 |

^1^The cyclic mimotope CQAFYASSPRKSIHIGAC was coated on streptavidin ELISA plates and binding of V3 mAbs was detected by alkaline phosphate-conjugated anti-human IgG (Fc) antibodies.
